# MST1 Suppression Reduces Early Brain Injury by Inhibiting the NF-*κ*B/MMP-9 Pathway after Subarachnoid Hemorrhage in Mice

**DOI:** 10.1155/2018/6470957

**Published:** 2018-06-19

**Authors:** Jie Qu, Hengli Zhao, Qiang Li, Pengyu Pan, Kang Ma, Xin Liu, Hua Feng, Yujie Chen

**Affiliations:** Department of Neurosurgery, Southwest Hospital, Third Military Medical University, Chongqing, China

## Abstract

**Background:**

Mammalian sterile 20-like kinase 1 (MST1), the key component of the Hippo-YAP pathway, exhibits an important role in the pathophysiological process of various neurological disorders, including ischemic stroke and spinal cord injury. However, during subarachnoid hemorrhage, the involvement of MST1 in the pathophysiology of early brain injury remains unknown.

**Methods:**

We employed intravascular filament perforation to establish the subarachnoid hemorrhage (SAH) mouse model. The MST1 inhibitor XMU-MP-1 was intraperitoneally injected at 1 h after SAH, followed by daily injections. MST1 in vivo knockdown was performed 3 weeks prior to SAH via intracerebroventricular injection of adeno-associated virus (AAV) packaged with MST1 shRNA. The SAH grade, behavioral deficits, TUNEL staining, Evans blue dye extravasation and fluorescence, brain water content, protein and cytokine expressions by Western blotting, immunofluorescence, and proteome cytokine array were evaluated.

**Results:**

Following SAH, the phosphorylation level of MST1 was upregulated at 12 h, with a peak at 72 h after SAH. It was colocalized with the microglial marker Iba1. Both XMU-MP-1 and MST1 shRNA alleviated the neurological deficits, blood-brain barrier (BBB) disruption, brain edema, neuroinflammation, and white matter injury, which were induced by SAH in association with nuclear factor- (NF-) *κ*B p65 and matrix metallopeptidase-9 (MMP-9) activation and downregulated endothelial junction protein expression.

**Conclusions:**

The current findings indicate that MST1 participates in SAH-induced BBB disruption and white matter fiber damage via the downstream NF-*κ*B-MMP-9 signaling pathway. Therefore, MST1 antagonists may serve as a novel therapeutic target to prevent early brain injury in SAH patients.

## 1. Introduction

Subarachnoid hemorrhage (SAH) is identified as a subtype of hemorrhage stroke; it is mainly caused by a ruptured aneurysm, with high mortality and morbidity worldwide [1]. Recent studies have indicated the pivotal role of the vascular neural network in the pathophysiological process of early brain injury after SAH [2]. The blood-brain barrier (BBB), identified as a component of this vascular neural network, is critical for brain homeostasis. After SAH, BBB disruption is an important pathological change, which contributes to brain edema, thus leading to poor outcomes [3]. Therefore, targeting BBB disruption may represent a prospective strategy to prevent early brain injury after SAH.

Mammalian sterile 20-like kinase 1 (MST1), a core component of the Hippo pathway, regulates multiple biological effects, including the response to oxidative stress, apoptosis, cell growth, and tumor suppression [4, 5]. In the central nervous system, many studies have demonstrated that oxidative stress-induced neuronal and neuroglia death is associated with MST1 activation [6–8]. In addition, MST1 exhibits fundamental functions in various central nervous system diseases, including vascular dementia, Alzheimer's disease, amyotrophic lateral sclerosis, and cerebral cavernous malformation [9–12]. Furthermore, genetic deletion of MST1 provides neuroprotection against ischemic stroke and spinal cord injury by attenuating neuronal apoptosis and the inflammatory response [13, 14].

MST1 has been shown to be upstream of nuclear factor- (NF-) *κ*B, a well-established conserved factor, for regulating neuroinflammation in many neurological diseases. However, the specific role of MST1 in the pathophysiological process of early brain injury remains elusive. Therefore, we aim to investigate the potential role of MST1 in a mouse model of SAH to determine its effects on early brain injury.

## 2. Materials and Methods

### 2.1. Experimental Animals

**Figure 1:**
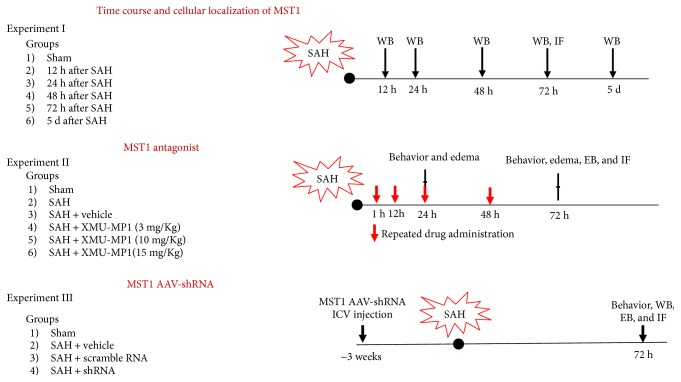
Brief illustration of the experimental design. WB: Western blot; EB: Evans blue extravasation; IF: immunofluorescence; ICV: intracerebroventricular.

Two hundred and sixty-one (261) male C57B6J mice, 23 g to 28 g in weight, were raised and housed by the Experimental Animal Center of Third Military Medical University (Chongqing, China). All animal experimental procedures were evaluated and approved by the Ethics Committee of Southwest Hospital, Third Military Medical University; the procedures were performed in accordance with the National Institutes of Health (NIH) Guide for the Care and Use of Laboratory Animals and are reported in accordance with the ARRIVE guideline. The mice were randomly allocated to different experimental groups as shown in Figure 1.

### 2.2. SAH Model

The mice underwent intravascular perforation procedures to mimic SAH as previously described [15]. Briefly, sodium pentobarbital (intraperitoneal injection, 40 mg/kg) was used to anesthetize the animals. The external carotid artery (ECA) was isolated and cut distally into a 2 mm stump. A 5-0 filament suture was subsequently threaded through the ECA to the inner carotid artery (ICA). The sharpened suture was pushed 2 mm further during resistance to penetrate the bifurcation of the anterior cerebral artery (ACA) and middle cerebral artery (MCA). The ICA reperfused after filament suture was subsequently withdrawn. Sham-operated mice underwent the same procedure, without perforating the cerebral artery.

Following euthanasia, the severity of the SAH grade was blindly evaluated as previously reported [16]. In general, the scores from 6 segments of the SAH were summed. Mice that exhibited a score < 8 with no obvious neurological deficits were excluded from the following experiments.

### 2.3. Adeno-Associated Virus (AAV) Vector Administration

Sodium pentobarbital (40 mg/kg, ip) was used to anaesthetize the mice. Three microliters of AAV (Vigene Biosciences Inc., Shandong, China) or AAV empty were intraventricularly administered into the lateral ventricles (0.33 *μ*l/min) using specific coordinates (0.5 mm posterior to bregma, 3 mm ventral to the skull, and 1 mm lateral to the sagittal line) and a stereotaxic frame. The SAH model was performed 3 weeks after AAV infusion. To improve the knockdown efficiency, four different shRNA duplexes were designed and mixed in the present study. Their sequences are provided in their 5′ → 3′ orientation:

shRNA1:

GCGGACCTGCATTATGGACAATCTCGAGATTGTCCATAATGCAGGTCCGTTTTT

shRNA2:

GCCAGGAATGTAACACGAAGTACTCGAGTACTTCGTGTTACATTCCTGGTTTTT

shRNA3:

GCCCGAATGTTGAGCGAGAATTCTCGAGAATTCTCGCTCAACATTCGGGTTTTT

shRNA4:

GCGTGAGAATTTCTGCCGGAATCTCGAGATTCCGGCAGAAATTCTCACGTTTTT

### 2.4. Neurological Scoring

The modified Garcia scale was employed to evaluate the neurological function at 24 and 72 h after SAH and included six measurements as follows: spontaneous activity, forepaw outstretching, symmetry of limb climbing, responses to body proprioception, and vibrissae touch [17]. For the Beam Balance Score text, the mice were placed on the center of a wooden beam to assess the walking distance within 1 min and were subsequently assigned 0–4 points in total. The neurologic scores determined by the two blinded observers were used to determine the grade.

### 2.5. Brain Edema

The brain edema of the mice was evaluated using a previously described method [15]. Under deep anesthesia, brain tissues were rapidly dissected and separated into two cerebral hemispheres and cerebellum on tin foil paper, and the wet weights of the specimens were determined. The brain specimens were subsequently incubated in a 75°C oven for 72 h, and the dry weights were recorded. The brain edema/brain water content was calculated as (wet weight − dry weight)/wet weight × 100%.

### 2.6. Evans Blue Dye Extravasation and Autofluorescence

As previously reported, the BBB permeability was evaluated by Evans blue dye extravasation and autofluorescence. Under deep anesthesia, Evans blue dye (2%, 5 ml/kg) was administered into the caudal vein. After 2 h, the mice were intracardially perfused with phosphate-buffered solution (PBS), and brain specimens were collected and rapidly weighed. Two milliliters of 50% trichloroacetic acid was added to the ipsilateral/right hemisphere, tightly homogenized, and subsequently centrifuged at 10,000 ×g for 30 min. The homogenates were then incubated overnight at 4°C. Supernatants that contained the Evans blue dye were collected following a subsequent centrifugation at 10,000 ×g for 30 min, and a spectrophotometer was used to measure the absorbance of the supernatant at 615 nm.

### 2.7. Immunofluorescence Staining

Immunofluorescence staining was performed at 72 h after the operation. Briefly, the mice were anesthetized and perfused with 4% paraformaldehyde. Brain specimens were rapidly collected and postfixed in 4% paraformaldehyde for 1 day and a 30% sucrose solution for the following 3 days. Fifteen *μ*m thick coronal sections were cut and treated with 0.3% Triton for 30 min. The brain sections were subsequently treated with 5% donkey serum for 1 h for antigen block and incubated with the primary antibodies overnight in a 4°C freezer, including anti-zonula occludens-1 (1 : 100, Invitrogen, Grand Island, NY), anti-von Willebrand factor (1 : 100 Abcam), anti-Iba1 (1 : 100, Abcam), and anti-MST1 (1 : 100, Invitrogen, Grand Island, NY), followed by FITC-conjugated secondary antibody (1 : 200, Beyotime Biotechnology, Shanghai, China) and cy3-conjugated secondary antibodies (1 : 200, Beyotime Biotechnology, Shanghai, China) for 1.5 h at room temperature. A confocal laser scanning microscope (Zeiss, Oberkochen, Germany) was used to detect the expression of fluorescent dyes.

### 2.8. Terminal Deoxynucleotidyl Transferase-Mediated dUTP Nick-End Labeling (TUNEL) Staining

The In Situ Cell Apoptosis Detection Kit I (Boster Biological Technology, Wuhan, China) was used to detect DNA fragmentation in the cell nuclei of brain specimen sections by following the operating manual. Sections were illustrated with DAB solution, and the apoptotic nuclei were identified by the presence dark-brown staining. TUNEL-positive cells were counted by two researchers in a blinded manner.

### 2.9. Western Blotting

The ipsilateral/right cortex was isolated and homogenized for Western blotting as previously described [15] with the following primary antibodies: anti-MST1 (1 : 1000, Cell Signaling Technology, Danvers, MA, USA), anti-phosphorylated MST1 (1 : 1000, Cell Signaling Technology, Danvers, MA, USA), anti-ZO-1 (1 : 500 Invitrogen, Grand Island, NY, USA), anti-occludin (1 : 1000 Abcam, Shanghai, China), anti-claudin-5 (1 : 1000, Abcam, Shanghai, China), anti-NF-*κ*B (1 : 1000, Cell Signaling Technology, Danvers, MA, USA), and anti-matrix metallopeptidase- (MMP-) 9 (1 : 1000, Cell Signaling Technology, Danvers, MA, USA). GAPDH (1 : 10,000, Proteintech, Rosemont, IL, USA) was used as an internal loading control. The relative density of the Western blots was normalized to the sham group.

### 2.10. Proteome Profiler Mouse Cytokine Array

Protein expression analysis of the sham group, MST1 shRNA group, and negative control group (sh-NC) was performed 72 h after SAH using the Proteome Profiler Antibody Arrays—Mouse Cytokine Antibody Array, Panel A (Catalog Number ARY006, R&D Systems, Minneapolis, MN, USA) according to the manufacturer's instructions as previously reported.

### 2.11. Statistical Analysis

All data are presented as the mean ± standard deviation (mean ± SD) and were analyzed using GraphPad Prism 6 (GraphPad Software, San Diego, CA). One-way ANOVA and Tukey's multiple comparisons were employed for comparisons among the different groups. The Kruskal-Wallis test was employed for the analysis of the behavior scores. *p* < 0.05 was considered statistically significant.

## 3. Results

### 3.1. Time Course of Endogenous P-MST1 and Phosphorylated-MST1 Expressions in Mice after SAH Injury

There were 6 mice (2 in the 12 h group, 1 in the 24 h group, 1 in the 48 h group, and 2 in the 5 d group); those with a score < 8 or no obvious neurological deficits were excluded from further analysis. And 12 mice (3 in the 12 h group, 2 in the 24 h group, 3 in the 48 h group, 2 in the 72 h group, and 2 in the 5 d group) that underwent SAH died due to severe hemorrhagic volume within 24 h of SAH. There is no significant difference in the mortality of each group (*p* > 0.05).

**Figure 2:**
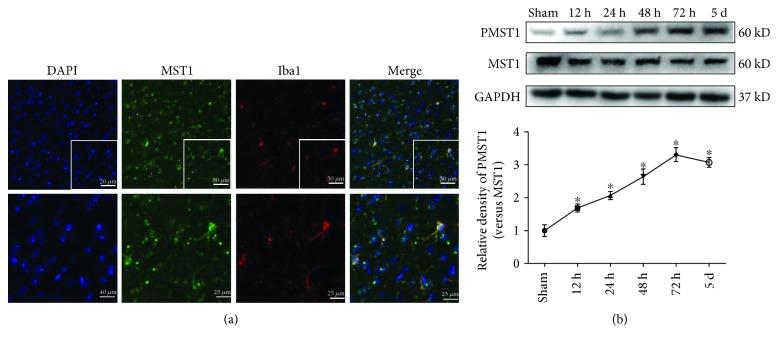
Time course of P-MST1 and MST1 expressions after SAH. (a) Representative images and Western blot analyses of P-MST1 and MST1 in the ipsilateral hemisphere in sham and SAH mice at 12 h, 24 h, 48 h, 72 h, and 5 days after SAH; *n* = 6 mice per group per time point. Relative densities were normalized against the densities in the sham group. ^∗^*p* < 0.05 versus the sham group. (b) Representative immunofluorescence staining slices of MST1 and Iba1 in the ipsilateral cortex at 72 h after SAH. Scale bar = 50 *μ*m (up) and 25 *μ*m (down).

The Western blot results indicated that the total protein expression of MST1 in the right/ipsilateral hemisphere decreased at 12 h after SAH and was subsequently maintained at a low level. Moreover, the phosphorylated MST1 increased at 12 h after SAH compared to the sham group, with a peak at 72 h (Figure 2(a)). Therefore, the tendency of the phosphorylation level of MST1 is the same as that of phosphorylated MST1. Furthermore, immunofluorescence images demonstrated that MST1 colocalized with microglia cells (Iba1) in the right cortex at 72 h after SAH (Figure 2(b)).

### 3.2. XMU-MP-1 Treatment Relieved Early Brain Injury after SAH

**Figure 3:**
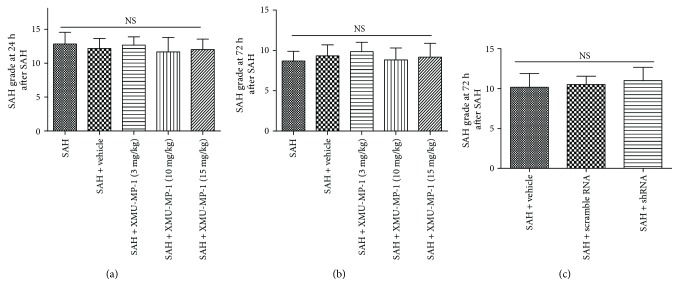
SAH grading scores of SAH mouse models in each group. The SAH grading scores of the SAH mouse models in experiment II at (a) 24 h and (b) 72 h after SAH, as well as in experiment III at 72 h after SAH.

No toxic effect of MST1 inhibition was identified in this study. The SAH grading score results showed that there were no significant differences among the experimental SAH groups at 24 h or 72 h (Figure 3). 4 mice (2 in the SAH group, 1 in the 3 mg/kg group, and 1 in the 10 mg/kg group) with a score < 8 as well as no obvious neurological deficits were excluded from further analysis, and 8 mice (1 in the SAH group, 2 in the vehicle group, 1 in the 3 mg/kg group, 2 in the 10 mg/kg group, and 2 in the 15 mg/kg group) that underwent SAH died due to severe hemorrhagic volume within 24 h of SAH. There is no significant difference in the mortality of each group (*p* > 0.05).

**Figure 4:**
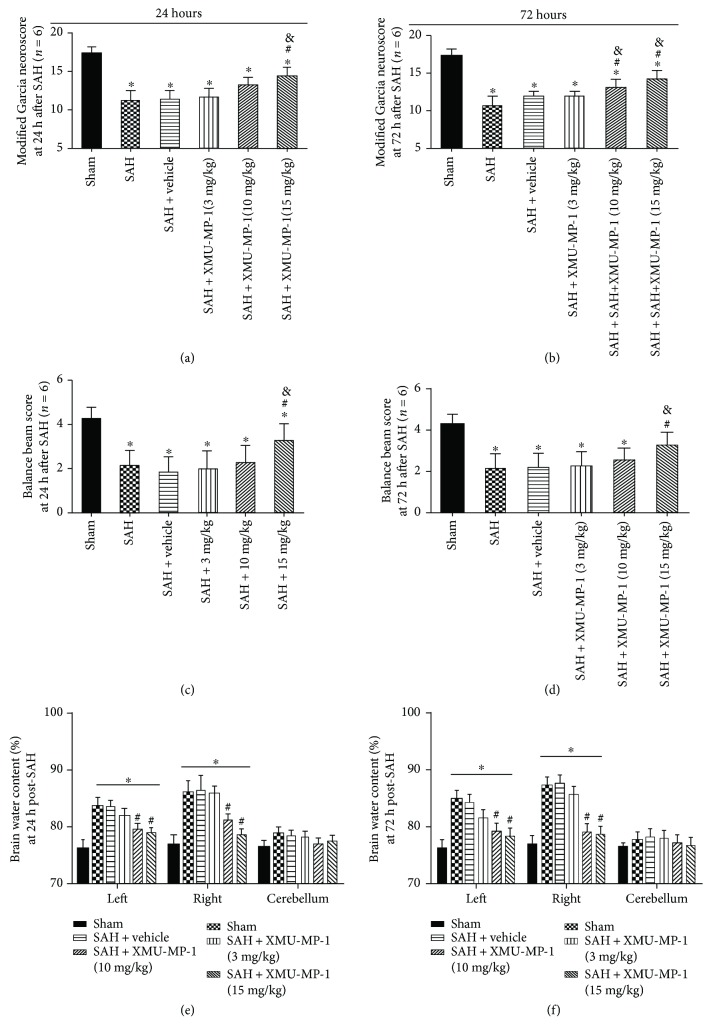
Effects of XMU-MP-1 treatment on early brain injury after SAH. Modified Garcia scores at (a) 24 h and (b) 72 h in each group after SAH. Beam balance scores at (c) 24 h and (d) 72 h in each group after SAH. Brain edema at (e) 24 h and (f) 72 h in each group after SAH (*n* = 6). ^∗^*p* < 0.05 versus the sham group; ^#^*p* < 0.05 versus the SAH group; ^&^*p* < 0.05 versus the SAH + vehicle group.

Both neurobehavioral scores were significantly lower in the SAH mouse model group than in the sham group at 24 h and 72 h after SAH. Compared with the vehicle treatment after SAH, MST1 inhibition improved the SAH-impaired neurological function on the modified Garcia test; both the 10 mg/kg dosage and the 15 mg/kg dosage of XMU-MP-1 treatments significantly improved the neurobehavioral outcomes at 72 h after SAH, whereas only the 15 mg/kg dosage exerted neuroprotective effects at 24 h after SAH (Figures 4(a) and 4(b)). Furthermore, the beam balance score indicated that the SAH mice exhibited substantially worse neurological deficits at 24 h and 72 h after SAH compared to the sham group, and the high dosage (15 mg/kg) XMU-MP-1 treatment remarkably improved neurological function (Figures 4(c) and 4(d)).

In addition, there was significantly greater brain edema in both hemispheres of the SAH mice than in the hemispheres of the sham-operated mice at 24 h and 72 h after SAH. Low concentrations (3 mg/kg) of XMU-MP-1 showed a tendency to alleviate this SAH-induced brain edema, whereas high concentrations (10 and 15 mg/kg) of XMU-MP-1 significantly decreased the SAH-induced brain edema compared to the vehicle treatment (Figures 4(c) and 4(d)).

**Figure 5:**
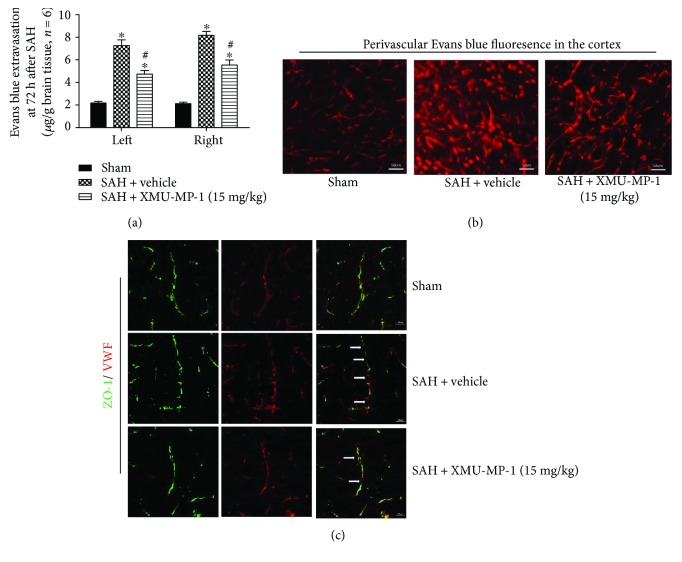
Effects of XMU-MP-1 administration on BBB integrity at 72 h after SAH. (a) Content of Evans blue extravasation at 72 h after SAH. (b) Representative Evans blue fluorescence images in the right/ipsilateral cortex at 72 h after SAH. (c) Representative immunohistochemistry images of ZO-1 and vWF at 72 h after SAH. White arrows indicate the disruption in continuous endothelial cells. ^∗^*p* < 0.05 versus the sham group; ^#^*p* < 0.05 versus SAH + vehicle. *n* = 6 per group. Scale bar = 50 *μ*m.

Compared with the brains of the sham-treated mice, the extravasated content of Evans blue was substantially higher in both hemispheres of the mouse model of SAH, and XMU-MP-1 (15 mg/kg) significantly alleviated the leakage (Figure 5(a)). Furthermore, Evans blue fluorescence in the ipsilateral cortex showed more dye leakage around the arteriole in the SAH mice than in the sham-treated mice, which was reduced by XMU-MP-1 treatment (Figure 5(b)). At 72 h after SAH, the continuous endothelial cells indicated by vWF-positive cells and the tight junctions indicated by ZO-1 structures were damaged. However, XMU-MP-1 treatment effectively reduced these disruptions (Figure 5(c)).

### 3.3. MST1 In Vivo Knockdown Improved Neurobehavioral Outcomes, Alleviated Neuroinflammation, and Reduced Evans Blue Extravasation at 72 h after SAH

The result of the SAH grading score showed that there was no significant difference among these experimental SAH (Figure 3(c)). 3 mice (2 in the SAH group and 1 in the shRNA group) with a score < 8 as well as no obvious neurological deficits were excluded from further analysis. And 4 mice (2 in the SAH group, 1 in the scramble RNA group, and 1 in the shRNA group) that underwent SAH died due to severe hemorrhagic volume within 24 h of SAH. There is no significant difference in the mortality of each group (*p* > 0.05).

**Figure 6:**
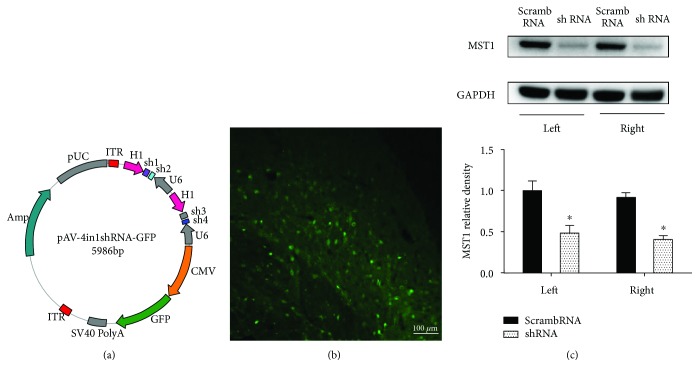
Infection and inhibition efficacy of the AAV. (a) The shRNA-containing vector. (b) Representative photo of the fluorescent tags (GFP). (c) Representative images and analysis by Western blot for the MST1 shRNA inhibitory effect. *n* = 6; ^∗^*p* < 0.05 versus scrambled shRNA. Scale bar = 100 *μ*m.

**Figure 7:**
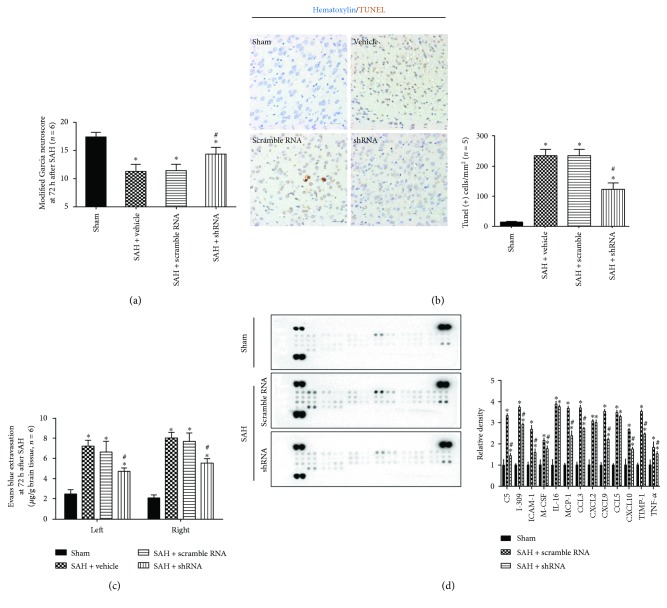
Effects of MST1 shRNA on neurobehavioral score, neural cell injury, Evans blue extravasation, and neuroinflammation. (a) Statistical analysis of the modified Garcia scores at 72 h after SAH. (b) Representative figures and statistical analysis of the cell counting of the TUNEL-positive cells at 72 h after SAH. (c) Statistical analysis of the Evans blue extravasation of each group at 72 h after SAH. (d) Representative bands and quantitative analyses of the proteome profiler mouse cytokine array in the indicated groups. Relative densities were normalized against the density in the sham group. *n* = 3; ^∗^*p* < 0.05 versus the sham group; ^#^*p* < 0.05 versus SAH + scramble RNA. Scale bar = 50 *μ*m.

To knock down MST1 protein expression, we used AAV to infect the whole brain (Figure 6(a)) and Western blotting to measure the infection and knockdown efficiency (Figures 6(b) and 6(c)). At 72 h after SAH, both the vehicle- and scrambled shRNA-pretreated SAH mice showed remarkable neurological deficits compared to the sham group; however, there was no significant difference between the two groups (*p* > 0.05). In the MST1 shRNA-pretreated group, the modified Garcia score was significantly improved (Figure 7(a)). In addition, compared with the sham group, there are much more TUNEL-positive cells in the cortex at 72 h after SAH (Figure 7(b)). In MST1 shRNA pretreatment group, the number of TUNEL-positive cells in the cortex was less than half of the vehicle group's (Figure 7(b)). Meanwhile, the MST1 shRNA-pretreated mice showed less Evans blue dye extravasation than both the vehicle- and scrambled shRNA-pretreated groups (Figure 7(c)). Based on the proteome profiler mouse cytokine array, the SAH groups exhibited 13 upregulated target proteins compared to the sham group. Among these upregulated proteins, cytokines (TNF-*α*) and chemokines (I-309, ICAM-1, MCP-1, M-CSF, CCL3, CXCL12, CXCL2, CXCL9, and CXCL10) were significantly downregulated by MST1 suppression compared with the expression in the negative control animals (Figure 7(d)).

### 3.4. Effects of MST1 Knockdown on p65 NF-*κ*B, MMP-9, and Tight Junction Protein Expression at 72 h after SAH

**Figure 8:**
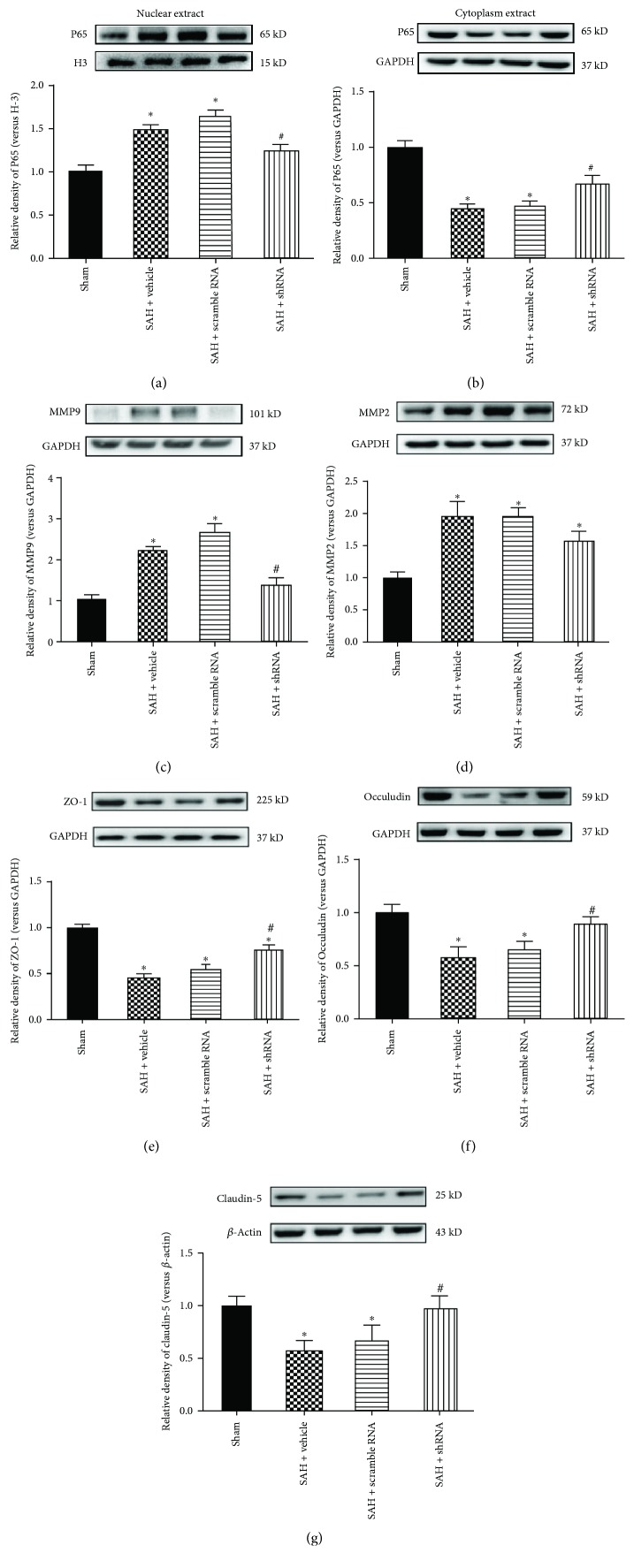
Western blot analysis of the effects of MST1 shRNA. Representative images and analysis of p65 NF-*κ*B protein expression in the (a) cytoplasm and (b) nucleus at 72 h after SAH. Representative images and analysis of (c) MMP-9 and (d) MMP-2 protein expressions at 72 h after SAH. (e–g) Representative images and analysis of ZO-1, occludin, and claudin-5 at 72 h after SAH. The band density values were normalized to the sham group. ^∗^*p* < 0.05 versus sham; ^#^*p* < 0.05 versus SAH + vehicle.

To evaluate the p65 NF-*κ*B activity, the cytoplasmic expressions and nuclear expressions of p65 NF-*κ*B were detected in each group. In the vehicle- and scrambled shRNA-pretreated SAH mice, the cytoplasmic expressions of p65 NF-*κ*B were significantly decreased and the nuclear expressions of p65 NF-*κ*B were significantly increased compared to the sham mice (Figures 8(a) and 8(b)). In addition, the MMP-2 and MMP-9 protein expressions were elevated in the two groups. However, compared to the SAH + vehicle group, MST1 shRNA pretreatment preserved the expression of p65 NF-*κ*B in the cytoplasm and decreased the nuclear expression of p65 NF-*κ*B (*p* < 0.05). Furthermore, MST1 shRNA downregulated the expression of MMP-9 but not MMP-2 (Figures 8(c) and 8(d)). The vehicle- and scrambled RNA-treated SAH mice also expressed significantly less ZO-1, occludin, and claudin-5 than did the sham mice. However, MST1 shRNA pretreatment upregulated the expressions of ZO-1, occludin, and claudin-5 (Figures 8(e)–8(g)).

### 3.5. MST1 Knockdown Reduces White Matter Fiber Damage at 72 h after SAH

**Figure 9:**
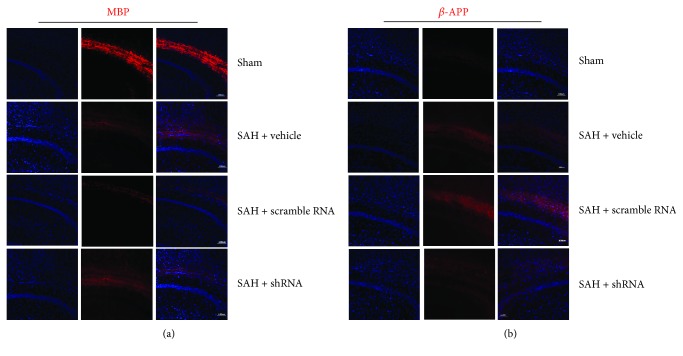
Effects of MST1 shRNA pretreatment on the white matter fiber damage induced by SAH. Representative immunofluorescence staining of MBP (a) and *β*-APP (b) in each group. Scale bar = 100 *μ*m.

To evaluate the white matter damage after SAH, immunofluorescent staining was used to detect the protein marker of white matter injury. Both the vehicle- and scrambled shRNA-pretreated SAH mice expressed significantly less myelin basic protein (MBP) than did the sham mice, and MST1 shRNA preserved the expression of MBP (Figure 9(a)) at 72 h after SAH. In addition, the amyloid precursor protein (APP) expression, a classic marker of axonal injury indicative of cytoskeletal damage, was significantly increased after SAH; however, MST1 shRNA pretreatment alleviated the APP expression (Figure 9(b)).

## 4. Discussion

In our study, we explored the time course of the MST1 phosphorylation level and cellular localization, as well as the function and mechanism of MST1 in the early brain injury during SAH. We determined that the phosphorylated MST1 expression was increased at 12 h after SAH compared to the sham group, with a peak at 72 h. It was colocalized with the microglia marker Iba1 in the cortex. A high dose of XMU-MP-1 (15 mg/kg), identified as an inhibitor of MST1/2, alleviated the neurological deficits, brain edema, and BBB disruption caused by SAH. In addition, to rule out nonspecific effects of the MST1 inhibitor, we also used shRNA (packaged by AAV) to in vivo knock down MST1; the downregulation of MST1 increased the neurological score and reduced the neuroinflammation, BBB disruption, and white matter injury. MST1 knockdown suppressed the NF-*κ*B-MMP-9 pathway, thus preserving the reduced tight junction proteins. In summary, these results supported our hypothesis that MST1 suppression could alleviate early brain injury in SAH mice by inhibiting the NF-*κ*B/MMP-9 pathway.

After subarachnoid hemorrhage, BBB disruption contributes to brain edema, followed by brain swelling, increased intracranial pressure, neuronal death, and neurologic deficits [3]. Previous studies have demonstrated that the inflammatory response may increase BBB permeability after SAH [16, 18, 19]. However, the involvement of MST1 and its associated pathological process is unknown. MST1, the mammalian Hippo ortholog, is the central component of the Hippo pathway [4]. Previous studies have suggested that MST1 activation contributes to neuronal apoptosis and inflammation. Genetic deletion of MST1 may reduce the inflammation and microglial activation after spinal cord injury and ischemic stroke [13, 14]. Consistent with these reports, we determined that pharmacological inhibition of MST1 or its knockdown alleviated the neurological deficits, brain edema, and BBB disruption induced by SAH. Furthermore, the expression of several proinflammatory cytokines was suppressed by MST1 inhibition, which is associated with NF-*κ*B inhibition. The activation of MMPs, particularly MMP-9, may contribute to the disruption of the BBB via degradation of tight junction proteins after SAH [16]. Our current results suggested that MST1 suppression repressed the MMP-9 expression, alleviated the degradation of tight junction proteins, and reduced the leakage of Evans blue dye. Moreover, we determined that the cytoplasm-nuclear translocation of p65 NF-*κ*B was substantially decreased by MST1 suppression.

MST1 has been associated with the immunological response in various systems. Our results demonstrated that increased MST1 contributes to the higher expressions of cytokines (TNF-*α*) and chemokines (I-309, ICAM-1, M-CSF, MCP-1, CCL3, CXCL12, CXCL2, CXCL9, and CXCL10), which play vital roles in neuroinflammation and early brain injury after SAH. Salojin et al. knocked out MST1 to alleviate experimental autoimmune encephalomyelitis and improve collagen-induced arthritis [20]. In addition, MST1-mediated dendritic cell- (DC-) dependent Th17 differentiation has been shown to regulate experimental autoimmune encephalomyelitis and antifungal immunity [21]. Kurz et al. reported that MST1-deficient (Mst1^−/−^) neutrophils were unable to migrate into inflamed tissues [22]. However, to date, the mechanisms by which MST1 and its downstream pathways affect neuroinflammation in acute central neural system injuries remain unknown and require further investigation.

After SAH, acute white matter injury is an important pathological process during early brain injury [23, 24]. Several recent studies have indicated that the activation of MMP-9 contributed to SAH-induced white matter injury. Genetic deletion of MMP-9 may alleviate the white matter damage observed after SAH [25]. Our studies also indicated a role of MST1 in activating MMP-9; therefore, we evaluated the effect of MST1 suppression on white matter after SAH. Consistent with previous studies, pretreatment with MST1 shRNA partially rescued the reduction in MBP (marker of myelin degradation) expression caused by SAH, whereas the elevated expression of *β*-APP (maker of axonal damage) induced by SAH was moderately inhibited by MST1 shRNA pretreatment. These results suggested that MST1 suppression protects nerve fibers and reduces brain edema after SAH.

The present study has several limitations. First, we investigated the short-term neuroprotective effects of MST1 suppression within 3 days after SAH. The long-term effects of MST1 suppression should also be examined in the future to further understand the MST1 involvement after SAH. In addition, XMU-MP-1 is an inhibitor of MST1/2 [26]. Although MST1 and MST2 have been shown to have the same effects in many studies [4, 27–29], they may not be equivalent in SAH. Thus, we cannot eliminate the possibility that MST2 suppression provided the neuroprotective effects for the early brain injury post-SAH. Finally, we explored the relation between MST1 and NF-*κ*B-MMP-9; however, how MST1 influences NF-*κ*B remains unknown. Thus, future studies should explore downstream of MST1.

## 5. Conclusions

In summary, our findings suggested that the serine/threonine kinase MST1 plays a pivotal role in the pathophysiological process of early brain injury after SAH. Inhibition of MST1 alleviated the neurological deficits, preserved the BBB integrity, reduced brain edema, and prevented white matter damage by inhibiting the downstream NF-*κ*B/MMP-9 signal after SAH. Therefore, MST1 suppression treatment may represent a promising therapeutic strategy for the future management of SAH patients.

## Data Availability

The data used to support the findings of this study are available from the corresponding author upon request.
