# Correction to “MST1 Suppression Reduces Early Brain Injury by Inhibiting the NF‐*κ*B/MMP‐9 Pathway after Subarachnoid Hemorrhage in Mice”

**DOI:** 10.1155/bn/9759567

**Published:** 2026-07-08

J. Qu, H. Zhao, Q. Li, et al., “MST1 Suppression Reduces Early Brain Injury by Inhibiting the NF‐*κ*B/MMP‐9 Pathway After Subarachnoid Hemorrhage in Mice,” *Behavioural Neurology*, 2018: 6470957, https://doi.org/10.1155/2018/6470957.

In the article, there is an error in Figure 9A, in which the SAH + Vehicle panels were duplicated in the SAH + shRNA panels due to a mistake introduced when preparing the figure. The correct Figure 9 is shown below:

**Figure 9:**
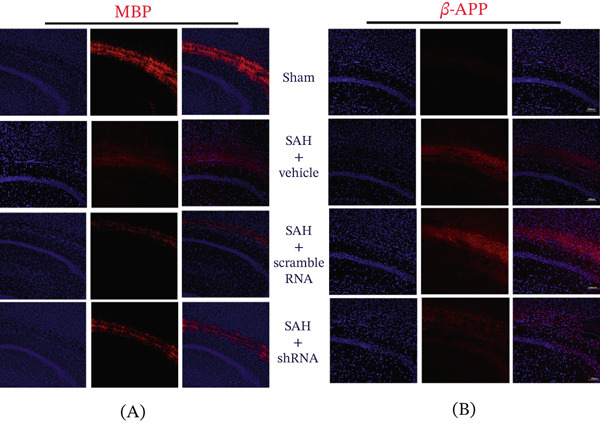
Effects of MST1 shRNA pretreatment on the white matter fiber damage induced by SAH. Representative immunofluorescence staining of (A) MBP and (B) β‐APP in each group. Scale bar = 100 μm.

We apologize for this error.

